# The Impact of a High-Fat Diet on Eye Health

**DOI:** 10.3390/nu17203271

**Published:** 2025-10-17

**Authors:** Kamila Pieńczykowska, Anna Bryl, Małgorzata Mrugacz

**Affiliations:** 1 Doctoral School, Medical University of Bialystok, ul. Jana Kilińskiego 1, 15-089 Bialystok, Poland; mynameiskama@gmail.com; 2 Department of Ophthalmology and Eye Rehabilitation, Medical University of Bialystok, 15-089 Bialystok, Poland; anna.bryl@umb.edu.pl

**Keywords:** lipids, high-fat diet, ocular health, retina, dry eye syndrome, cataract, age-related macular degeneration, fatty acids, myopia, glaucoma

## Abstract

**Background**: The increasing prevalence of high-fat diets is associated with a rise in metabolic and neurodegenerative diseases. The retina and retinal pigment epithelium are metabolically active tissues exposed to oxidative stress, making them particularly vulnerable to lipid excess. **Materials and Methods**: A systematic literature review was conducted covering years until 2025 inclusive. **Results**: High-fat diets lead to cholesterol accumulation and lipid metabolism disturbances in the retina, retinal pigment epithelium, and ocular vessels. They activate inflammatory and oxidative stress pathways, resulting in structural and functional damage. Omega-3 deficiency exacerbates inflammation, while supplementation improves the tear film stability, corneal epithelial function, intraocular pressure regulation, and exerts neuroprotective effects. **Conclusions**: High-fat diets represent a significant risk factor for ocular diseases by disrupting lipid metabolism, enhancing inflammation, and inducing oxidative stress. Omega-3 fatty acid supplementation reduces inflammation and supports ocular functions.

## 1. Introduction

**Figure 1:**
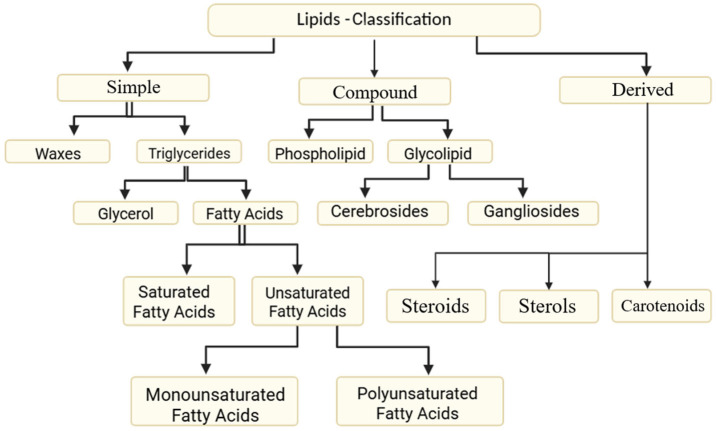
Classification of lipids.

The rapid global shift toward high-fat dietary patterns has coincided with a surge in metabolic and degenerative diseases, yet the ocular consequences of such diets remain underrecognized. The retina and retinal pigment epithelium (RPE) are metabolically active tissues with high lipid content and continuous oxidative stress exposure, making them particularly vulnerable to dietary lipid excess. A high-fat diet (HFD) results in significantly elevated cholesterol levels in the retina, RPE, liver, and serum, accompanied by a marked reduction in the expression of cholesterol metabolism–related genes in these tissues, compared to a normal diet [1]. A high-fat diet is a dietary regimen in which a large proportion of total calories comes from fats, often exceeding 30–35% of daily caloric intake. An example of such a diet, which has gained in popularity due to possibility of rapid weight loss, is the ketogenic diet. Typically, the diet’s macronutrient ratio consists of about 5% carbohydrates, 75% fats, and 20% proteins [2]. Fatty acids represent the fundamental constituents of lipids (fats) and are structurally composed of a hydrocarbon chain with a terminal carboxyl group (−COOH) at one end and a methyl group (−CH_3_) at the other, connected by repeating methylene units (−CH_2_−). Based on the absence or presence of double bonds, fatty acids are categorized into saturated fatty acids (SFA) and unsaturated fatty acids, respectively (Figure 1). The harmful effects of SFA are driven by several mechanisms: buildup of diacylglycerol and ceramide, activation of NF-κB, protein kinase C, and mitogen-activated protein kinases, leading to increased inflammatory gene expression in white adipose tissue, immune cells, and myotubes, reduced PPARγ coactivator-1α/β activity and lower adiponectin levels, impairing glucose and fatty acid oxidation, and recruitment of immune cells, including macrophages, neutrophils, and bone marrow-derived dendritic cells, to white adipose tissue and muscle [3]. Among unsaturated fatty acids, the classification into omega-6 and omega-3 families depends on the position of the first double bond counted from the methyl end of the carbon chain. The omega-6 fatty acid family includes linoleic acid, γ-linolenic acid, dihomo-γ-linolenic acid, and arachidonic acid. Conversely, the omega-3 fatty acid family encompasses α-linolenic acid, eicosapentaenoic acid, and docosahexaenoic acid (DHA). These structural and biochemical distinctions are critical, as they underlie the functional diversity of fatty acids in physiological and pathological processes, including their influence on ocular health through roles in retinal structure, inflammatory regulation, and intraocular pressure modulation [4]. Omega-3–derived epoxyeicosanoids exhibit cardioprotective, vasodilatory, anti-inflammatory, and anti-allergic activities, which underlie the health-promoting effects of *n*-3 long-chain polyunsaturated fatty acids (LC-PUFAs) across a range of pathological conditions, including cardiovascular disease, bronchial disorders, intraocular neovascularization, allergic intestinal inflammation, and inflammatory pain [5].

Defining the molecular and cellular consequences of high-fat dietary intake on the eye is essential for identifying early biomarkers and therapeutic targets. This review synthesizes current experimental and clinical evidence on HFD-induced retinal and RPE injury, highlighting the interplay between lipid metabolism, inflammation, and neurodegeneration in the pathogenesis of vision loss. While preclinical evidence clearly demonstrates lipid accumulation and metabolic dysfunction in ocular tissues following high-fat feeding, most findings derive from animal models using extreme dietary conditions not representative of human nutrition. Translational relevance and long-term human outcomes therefore remain uncertain.

## 2. Materials and Methods

**Figure 2:**
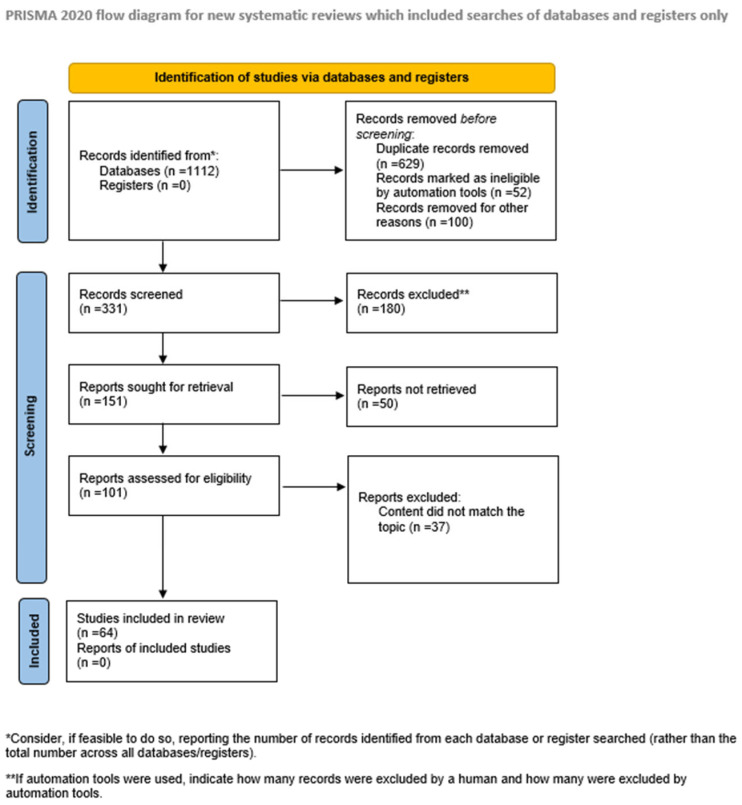
The PRISMA flowchart.

The focus of this study was to explain the connection between high-fat diet and the risk of developing eye diseases. Therefore, an extensive review of the literature was undertaken from June 2025 to September 2025, drawing on established databases such as PubMed, Scopus, and Web of Science to locate pertinent studies. Only studies published in English were included, while geographic origin was not considered a criterion for inclusion. Our selection prioritized recent literature, particularly from the past five years, unless older studies offered novel or significant insights. All study types were considered. Studies were excluded if the full text was not available online, if they were published in languages other than English, or if they were conference abstracts, posters, or unrelated to the research question. Finally, we have selected 64 articles to include in this review. The review protocol was registered with Prospero (PROSPERO CRD1167766). The conduct and reporting of this systematic review adhered to the PRISMA statement and its extension for network meta-analyses. The workflow for the identification and stepwise selection of the studies is presented in Figure 2.

## 3. Impact of High-Fat Diet on Retinal Structure and Function

### 3.1. Retinal Structure

The retina constitutes the innermost layer of the eye and is essential for visual processing, converting photon-derived light energy into complex three-dimensional visual representations. It is structurally organized into ten well-defined layers, arranged sequentially from the innermost aspect adjacent to the vitreous chamber to the outermost interface with the choroid:

- **Inner Limiting Membrane (ILM)**—The ILM constitutes the basal boundary of the retina, interfacing with the vitreous body. It primarily comprises the endfeet of Müller glial cells, which contribute to retinal homeostasis by maintaining structural organization and supporting neuronal function.
- **Retinal Nerve Fiber Layer (RNFL)**—This layer consists of unmyelinated axons of retinal ganglion cells (RGCs), interspersed with astrocytes and Müller cell processes. The ILM serves as the basement membrane for this layer, providing structural anchoring.
- **Ganglion Cell Layer (GCL)**—Contains the somata of RGCs, whose axons converge to form the optic nerve, facilitating the transmission of visual information to central targets.
- **Inner Plexiform Layer (IPL)**—A synaptic layer where bipolar cell axons contact ganglion cell dendrites. Amacrine cells also form synapses here, playing a critical role in modulating signal transmission through lateral inhibition and temporal filtering.
- **Inner Nuclear Layer (INL)**—Composed of the cell bodies of bipolar, horizontal, and amacrine cells. Bipolar cells act as intermediaries, relaying signals from photoreceptors to ganglion cells, while horizontal and amacrine cells provide lateral modulation of synaptic input.
- **Outer Plexiform Layer (OPL)**—The site of synaptic interactions between photoreceptor terminals and the dendrites of bipolar and horizontal cells, enabling vertical and horizontal signal integration.
- **Outer Nuclear Layer (ONL)**—Contains the nuclei of rod and cone photoreceptors, which are responsible for phototransduction.
- **External Limiting Membrane (ELM)**—Composed of adherens and gap junctions between photoreceptors and Müller cells. This layer demarcates the boundary between the nuclear components of photoreceptors and their inner segments.
- **Photoreceptor Layer (PRL)**—Comprises the inner and outer segments of rods and cones. The outer segments contain stacks of membranous discs enriched with opsins (e.g., rhodopsin), which is essential for light absorption, while the inner segments house mitochondria that support the high metabolic demands of phototransduction.
- **Retinal Pigment Epithelium (RPE)**—A monolayer of pigmented epithelial cells located between the neural retina and Bruch’s membrane. The RPE performs multiple functions, including forming part of the blood-retinal barrier (in concert with retinal endothelial cells), recycling visual pigments (conversion of all-trans-retinal to 11-cis-retinal), phagocytosing shed photoreceptor outer segments, and secreting trophic factors essential for retinal and choroidal homeostasis [6].

### 3.2. High-Fat Diet and Inflammatory Pathways in Retina (Figure 3)

Long-term exposure to a high-fat diet (HFD) profoundly reshapes retinal lipid composition, characterized by elevated levels of cholesteryl esters (CE), phosphatidylcholine (PC), and phosphatidylglycerol (PG), alongside reduced eicosanoids. Proteomic profiling indicated hyperactivation of the primary bile acid biosynthesis pathway in HFD retinas. Single-cell transcriptomics further revealed cell type–specific transcriptional changes, with distinct regulatory responses in Müller glia (MG) and rod photoreceptors. Notably, the upregulation of bile acid synthesis was largely confined to MG cells and likely involved alternative metabolic routes, highlighting their pivotal role in retinal lipid regulation under high-fat conditions [7]. HFD also causes endothelial dysfunction in the ophthalmic artery. This effect appears to be driven by increased expression of RAGE, but not LOX-1, along with the upregulation of the pro-oxidant enzyme NOX2, which likely promotes reactive oxygen species (ROS) production and contributes to the vascular impairment [8]. Apolipoprotein E (ApoE) plays a key role in removing cholesterol-rich lipoproteins from circulation. In ApoE gene knockout models fed an HFD, atherosclerosis and lipid metabolism abnormalities are aggravated, intensifying age-related retinal structural damage and intraocular microcirculation abnormalities. These changes are accompanied by increased expression of the pro-inflammatory cytokine TNF-α, inducing microenvironmental inflammation and leading to functional impairment of the retina [9]. Peroxisome proliferator-activated receptor γ coactivator 1α (PGC-1α) plays a critical role in maintaining RPE metabolic balance, regulating lipid droplet formation, supporting mitochondrial function, and protecting against oxidative stress. In mice, PGC-1α downregulation led to RPE and retinal dysfunction, along with lipid deposits, as evidenced by ERG and funduscopy. In vitro, suppression of PGC-1α in RPE cells disrupted lipid metabolism and fatty acid β-oxidation caused lipid droplet buildup, lowered triglyceride synthesis, and promoted cholesteryl ester conversion. Deficiency in PGC-1α also impaired LDL uptake and cholesterol efflux while upregulating HMGCR activity, thereby stimulating cholesterol biosynthesis. Additionally, it downregulated genes linked to mitochondrial biogenesis, dynamics, and activity, reduced mitochondrial potential and antioxidant defenses, and increased RPE vulnerability to oxidative stress and lipid-peroxidation-induced cell death. Therefore, PGC-1α inhibition promotes AMD-like changes in the RPE and drives retinal degeneration through disturbed lipid metabolism [10]. Suppression of the Pgc-1α gene, together with aging and environmental factors like high-fat diet, can trigger AMD-like features in mice. These include accumulation of lipofuscin, deposits in the basal and outer collagenous layers, thickened Bruch’s membrane with CML-containing deposits, enlarged blood vessels, and loss of choriocapillaris endothelial fenestrations. The mice also display RPE and photoreceptor degeneration, upregulation of drusen-associated genes and Vegfa, reduced antioxidant defenses and mitochondrial function, and impaired autophagy in the RPE and retina [11].

## 4. The Role of Fats in Myopia

Elevated intake of saturated fat and cholesterol was positively associated with increased axial length in a study conducted by Lim et al. [12]. In children aged 5–12 years, those with myopia showed a significantly lower mean daily intake of fat, omega-3 fatty acids, and retinol compared to their emmetropic and hyperopic peers [13]. In guinea pigs and mice, omega-3 PUFAs were shown to slow myopia progression. They also counteracted form-deprivation (FD)-induced alterations in choroidal structure, vasculature, and scleral HIF-1α protein levels. These omega-3 PUFA-mediated effects may play a key role in preventing scleral myofibroblast transdifferentiation and preserving normal extracellular matrix remodeling, thereby protecting against myopia development [14]. In a study by Zhang et al., reduced dietary intake of omega-3 PUFAs was significantly linked to myopia and greater axial length elongation, suggesting a potential protective effect of omega-3 PUFAs against myopia development in children. Oppositely, higher intake of saturated fats was correlated with increased axial length [15]. As proved by Xue et al., higher concentrations of omega-3 fatty acids, docosahexaenoic acid (DHA), the DHA-to-total fatty acid ratio, the polyunsaturated fatty acid (PUFA)-to-total fatty acid ratio, the PUFA-to-monounsaturated fatty acid ratio, and the overall degree of unsaturation demonstrated nominal positive associations with greater choroidal thickness [16]. Similar conclusions come from Mendelian randomization and cross-sectional studies by Lu et al., which indicates that higher levels of omega-3 PUFAs, particularly DHA and EPA, may reduce the risk of high myopia. Genetic analyses further implicate retinal FADS1 expression, suggesting a tissue-specific protective mechanism, though additional studies are needed for confirmation [17]. These findings suggest that a higher systemic availability of omega-3 fatty acids and favorable fatty acid composition may contribute to maintaining or enhancing choroidal thickness, potentially supporting a protective role in ocular health. Although several cross-sectional and animal studies suggest that omega-3 fatty acids may protect against axial elongation and myopia progression, human evidence remains limited and partly inconsistent. Observational designs preclude causal inference, and dietary assessments often rely on self-reported intake. Randomized trials evaluating omega-3 supplementation and choroidal structural outcomes are lacking, representing a major gap in the field.

## 5. Dry Eye Syndrome and High Fat Diet

A population-based clinical study conducted in Korea discovered a link between dry eye and elevated serum cholesterol levels in women—the risk of developing DED was 77% higher in a group of women with a high cholesterol level. A significantly lower percentage of low HDL levels was observed in females diagnosed with DED and experiencing its symptoms compared to the control group [18]. According to Wu et al., a high-fat diet causes clear damage to the ocular surface, such as reduced tear production, prominent ocular surface staining, and a marked loss of goblet cells. It also leads to impaired corneal epithelial barrier function and significant squamous metaplasia in both the corneal and conjunctival epithelia. Additionally, the HFD increases the expression of critical regulators of oxidative stress and promotes cell apoptosis in ocular surface epithelial cells [19]. Cai et al. proved that the HFD successfully established a mouse model of dry eye disease. Significant lipid buildup was observed in the meibomian and lacrimal glands, indicating effective modeling of hyperlipidemia. HFD feeding led to progressive meibomian gland (MG) opening obstruction, lid margin depigmentation, and thick, milk-like meibum. Tear secretion declined after 8 weeks, and tear film stability (TBUT) decreased by 16 weeks, showing impaired lacrimal gland function. All these changes happen because a high-fat diet promotes macrophage polarization toward the M1 phenotype, which subsequently triggers inflammation [20]. A shortage of omega-3 lipids in the tear film may contribute to persistent ocular surface inflammation in dry eye disease. Patients who took omega-3 supplements showed increased PUFA levels in their tear film and a reduced, less inflammatory omega-6:omega-3 ratio. This indicates that orally consumed omega-3 supplements are bioavailable enough to directly influence inflammatory lipid profiles in the human tear film [21]. It was demonstrated that omega-3 and omega-6 can act directly on immortalized human meibomian gland epithelial cells to influence the quality and quantity of intracellular lipids by inducing the accumulation of small neutral lipid-containing vesicles, but not lysosomes. The vesicular response was linked to elevated intracellular triglyceride levels. Expression of free or esterified cholesterol, as well as phospholipids, was not altered by exposure to the fatty acids. Moreover, proliferation of human meibomian gland epithelial cells was suppressed by these fatty acids, either individually or in combination, in serum-free but not in serum-containing media [22]. Dietary supplementation with essential fatty acids and antioxidants has been shown to effectively alleviate all hallmark symptoms of dry eye (such as scratchiness, stinging, redness, grittiness, and blurred vision). Additional benefits included reduced reliance on artificial tears, diminished conjunctival hyperemia, and enhanced tear film function [23]. In cases where standard therapy with artificial tears is insufficient, supplementation with 1.5 g of omega-3 fatty acids may serve as a beneficial adjunct treatment, as this dose can improve tear breakup time in individuals with meibomian gland dysfunction (MGD) [24]. Neuroprotectin D1, a specialized pro-resolving mediator derived from DHA, was identified in the tissue of animal eyes with dry eye after corneal surgery, and DHA was shown to promote the nerve-regenerative activity of pigment epithelial-derived growth factor, while also suppressing the production of the pro-inflammatory mediator leukotriene B4 [25,26]. Omega-3 and omega-6 fatty acid supplementation may also lower the expression of the conjunctival inflammatory marker HLA-DR, which is normally limited to professional antigen-presenting cells; however, in dry eye disease, it is abnormally expressed on conjunctival epithelial cells, where its levels rise with disease severity [27,28]. The omega-3 index may serve as a predictor for identifying dry eye patients who are most likely to respond to oral omega-3 supplementation [29]. A study by Park et al. showed that re-esterified triglyceride-type omega-3 supplementation had beneficial effects in treating non-specific dry eye following uncomplicated cataract surgery. Oral omega-3 improved patients’ subjective symptoms, while objective inflammatory markers on the ocular surface, including the Oxford score and MMP-9 levels, were reduced after supplementation [30]. Similar conclusions were drawn by Hong et al., where the rTG-omega-3 group showed significant improvements in TBUT, corneal fluorescein staining, and strip meniscometry tube scores compared to the control group. Also, meibomian gland quality and expressibility showed significant improvement in patients with severe MGD [31]. Oral omega-3 fatty acids supplementation after LASIK may improve tear secretion but does not enhance tear film stability—the tear breakup time (TBUT) decreased after LASIK in both treatment and control groups, with no benefit from omega-3 fatty acids. In contrast, Schirmer scores (tear secretion) were significantly higher in the omega-3 fatty acids group at 3 months, suggesting a potential effect on lacrimal function, though both groups remained above the dry eye diagnostic threshold. Ocular Surface Disease Index (OSDI) scores and conjunctival staining showed minimal clinical significance, though fewer omega-3 fatty acids patients had conjunctival staining [32]. While both experimental and clinical findings indicate that high-fat diets can aggravate ocular surface inflammation and that omega-3 supplementation may mitigate symptoms, results are heterogeneous. Trials differ in omega-3 source, dose, duration, and outcome measures, leading to variable conclusions. Some large randomized studies report only modest or nonsignificant improvements. Future investigations should standardize endpoints, explore mechanistic biomarkers, and stratify participants by baseline lipid profile or omega-3 index.

## 6. The Impact of Lipids on Age-Related Macular Degeneration (AMD)

Age-related macular degeneration represents a major cause of irreversible vision loss in the elderly, contributing to roughly 8.7% of global visual impairment and expected to impact nearly 288 million individuals worldwide by 2040 [33]. AMD is categorized into early and late stages, both leading to central vision loss that primarily affects the macular region of the retina. The early stage is defined by the appearance of soft drusen deposits or pigmentary changes in the retina, whereas the late stage is distinguished by advanced manifestations such as geographic atrophy or choroidal neovascularization. Studies have shown that the risk of developing AMD is closely associated with a combination of genetic susceptibility, environmental exposures, aging, lifestyle habits, and cardiovascular health [34]. Likewise, obesity, recognized as a major contributing factor, is shaped by metabolic, genetic, behavioral, and environmental influences [35]. Studies have shown that patients with AMD frequently exhibit hyperlipidemia and lipid accumulation in drusen within the choroid [36]. A high-fat diet promotes amyloid-beta (a major constituent of drusen) buildup in the retinal pigment epithelium (RPE) of aged TgAPPswePS1 transgenic mice, resulting in RPE damage, with pyroptosis of RPE cells potentially serving as a key contributing factor. Pyroptosis, a distinct form of programmed cell death, is initiated through activation of the inflammasome—a protein complex comprising NLRP3 and pro-caspase-1. In this study, NLRP3 and caspase-1 immunoreactivity was minimal in the control and Tg groups but markedly elevated in RPE cells of the fat group. Pyroptosis is marked by cell swelling, plasma membrane pore formation, and the subsequent release of pro-inflammatory cytokines IL-1β and IL-18, which levels were elevated in the fat group [37]. A recent study in male Chinchilla rabbits fed a high-fat, high-sucrose diet (HFSD) introduced a promising dry AMD model, especially for reticular pseudodrusen (RPD)-like lesions. The animals developed dome-shaped, hyper-reflective lesions between the ellipsoid zone and RPE, composed of enlarged lipid droplets around RPE cells, likely due to impaired visual cycle processes from downregulated retinol metabolism genes (RPE65, LRAT). These droplets may act as retinosomes but also disrupt RPE function, marking early AMD pathology. HFSD rabbits also showed retinal degeneration—reduced ERG responses, photoreceptor loss, gliosis, and vascular rarefaction—along with elevated ocular complement factor C3, indicating complement activation linked to AMD [38]. RPE and choroidal tissues of mice fed a high-fat diet for 4 months also had more than double the lipid levels of controls. Retinal imaging revealed marked lipid droplet accumulation across retinal layers, and fundoscopy confirmed deposits resembling drusen in AMD. Lipid droplet accumulation was accompanied by significant RPE impairment, an early feature of AMD. The RPE showed loss of normal cuboidal shape, increased multinuclear cells, disrupted CTNNB1 localization, and loss of basal infoldings. Ultrastructural analysis revealed vacuoles, lipid granules, undigested photoreceptor segments, and swollen mitochondria. Key RPE markers—RPE65, KRT18, and MERTK—were also markedly reduced, indicating compromised RPE structure and function [39]. Reticular pseudodrusen (RPD) are less frequent in patients taking lipid-lowering drugs, suggesting statins may reduce RPD risk by modulating lipoprotein function. These results emphasize the role of retinal lipid trafficking in RPD formation and are supported by preliminary analyses of plasma fatty acid profiles in relation to different drusenoid deposits in age-related maculopathy [40]. Higher intake of omega-3 PUFAs, along with regular consumption of fish and nuts, may help reduce the risk of developing early AMD. Evidence also suggests possible interactions between fish, nut, or long-chain omega-3 PUFA intake and other factors—such as smoking, omega-6 PUFA consumption, beta-carotene intake, and the serum total cholesterol to HDL-C ratio—in influencing the AMD risk [41]. Greater consumption of DHA + EPA (particularly DHA) and fatty fish was linked to a 17–40% reduced risk of visually significant intermediate AMD, but showed no effect on the risk of advanced AMD [42]. Evidence linking high-fat intake to AMD is biologically plausible but not fully consistent. Differences in dietary patterns, genetic background, and lipid-lowering drug use complicate interpretation. Some epidemiological studies suggest protective associations with moderate PUFA or fish consumption, while others show no effect or even increased risk at high intakes. Moreover, many animal studies employ high-fat, high-sucrose diets that may simultaneously model obesity and insulin resistance, confounding lipid-specific effects. Controlled human studies isolating the impact of dietary fat composition on early AMD biomarkers are urgently needed.

## 7. The Connection Between Cataract and HFD

Serum LDL-C and TG concentrations demonstrated a significant association with age-related cataract, whereas no statistically significant variation in serum lipid profiles was observed among nuclear, cortical, and posterior subcapsular cataract subtypes [43]. Elevated intake of total fat and cholesterol was associated with an increased risk of cataract overall and all its subtypes. There can also be a statistically significant positive correlation between meat consumption and cataract occurrence [44]. Similar conclusions can be drawn from a study conducted in Iran—people with cataract tend to have higher scores of total fat and saturated fatty acids in their diet than healthy ones [45]. PUFA intake exhibited a significant positive association with the prevalence of nuclear opacity—particulary the 18-carbon PUFAs: linoleic acid and linolenic acid [46]. Although several studies associate higher fat and cholesterol intake with increased cataract risk, most are cross-sectional and subject to confounding by age, metabolic status, and comorbidities. The biological mechanisms linking dietary lipids to lens opacification remain speculative, with limited mechanistic data on oxidative or osmotic pathways. Interventional evidence demonstrating causality is currently lacking.

## 8. The Association Between Glaucoma and HFD

Consumption of high-fat, high-calorie diets is associated with an elevated risk of glaucoma, mainly normal tension glaucoma [47]. López-Gil et al. found that high consumption of ultra-proccessed food, which is rich in saturated and trans fats, elevates the risk of developing glaucoma [48]. Alternatively, a low-fat diet enriched with vegetables, fruits, and grains was not associated with a reduction in the incidence of primary open-angle glaucoma (POAG). Moreover, among individuals with the lowest baseline fat intake, further reductions in dietary fat appeared to increase the risk of developing POAG [49]. Dietary fat may modulate intraocular pressure (IOP) via mechanisms involving endogenous *n*-6 prostaglandins, and evidence indicates that fat-free diets, as well as parenteral nutrition, can lead to a reduction in IOP [50]. Dietary fat intake seems to influence the levels of intraocular prostaglandins by competing for the ocular enzymes responsible for producing arachidonic acid—the precursor of prostaglandin F2α—thereby modifying IOP in a manner that could affect the risk of developing POAG [51]. An elevated dietary *n*-3/*n*-6 polyunsaturated fat ratio has been associated with an increased risk of POAG, particularly the high-tension subtype. This connection can be observed especially among older patients [52]. However, increased dietary *n*-6 fatty acid intake alone may elevate *n*-6 prostaglandin levels (e.g., prostaglandin F2α), helping maintain IOP at less harmful levels and potentially lowering POAG risk [53]. According to Edokpa et al., higher consumption of meat, fish, and vegetable fats was linked to a lower risk of developing glaucoma [54]. In contrast, an energy-restricted diet high in salmon, a key source of omega-3 fatty acids, was associated with reduced serum levels of prostaglandin F2α [55]. Similar findings were drawn by Kinouchi et al.—greater weekly meat consumption has been inversely associated with the prevalence of OAG among Japanese women [56]. Low intake of fatty fish or walnuts was linked to a higher risk of POAG. These findings indicate that omega-3 fatty acids may play a protective role in POAG, potentially through their vascular and neuroprotective properties [57]. Omega-3 fatty acid intake was found to lower intraocular pressure. Experimental findings indicated that omega-3 supplementation enhanced aqueous humor outflow, thereby leading to a reduction in intraocular pressure in mice [58]. Three months of oral omega-3 supplementation significantly lowered IOP in human adults with normal blood pressure [59]. Adequate omega-3 fatty acid intake has been associated with a reduced risk of glaucoma among individuals aged 60 years and older; thus, ensuring sufficient consumption of omega-3 fatty acids is recommended in this population [60]. Supplementation with omega 3-PUFAs, whether used alone or together with timolol, helps preserve retinal ganglion cells in the mouse model of hereditary glaucoma. The combination of omega 3-PUFAs and timolol provides stronger neuroprotection than either treatment on its own. These findings highlight the contribution of inflammation to glaucoma pathogenesis and suggest that omega 3-PUFA supplementation may partly aid in modulating retinal inflammation. However, since inflammation was not fully suppressed by the combined treatment, it is likely that mechanisms beyond the downregulation of IL-18 and TNF-α also play a role in omega 3-PUFA-mediated neuroprotection in the retina [61]. Omega-3 fatty acids in phospholipid form are able to cross the blood–retinal barrier and RPE directly through the Mfsd2a transporter, achieving high concentrations within the eye. In contrast, omega-3 fatty acids in triglyceride, free fatty acid, or ethyl ester forms—commonly found in fish oil supplements—cross the barrier primarily by passive diffusion, which reduces their bioavailability [62]. In glaucoma, oxidative stress contributes to apoptosis, cytoskeletal changes, extracellular matrix accumulation, and cellular senescence in trabecular meshwork cells. Omega-3 preserves metabolic activity, reduces cytoskeletal stress fibers, and modulates certain extracellular matrix and inflammatory responses, while omega-6 suppresses proliferation, increases fibronectin and connective tissue growth factor, and mitigates some oxidative stress, though it induces inflammatory responses [63]. Eicosapentaenoic acid (EPA) and docosahexaenoic acid (DHA) have been shown to exert beneficial effects on systemic microcirculation, enhance ocular blood flow, and contribute to the reduction in optic neuropathy [64]. Higher daily intake of the omega-3 PUFAs EPA and DHA was linked to a reduced risk of developing glaucomatous optic neuropathy. In contrast, greater overall consumption of total PUFAs (including both omega-3 and omega-6 types) in the upper intake quartiles was associated with an increased risk of glaucoma [58]. The relationship between dietary fat composition and POAG risk remains complex and nonlinear. While some studies suggest that excessive or imbalanced PUFA intake—particularly a high *n*-3/*n*-6 ratio—may increase glaucoma risk, others demonstrate IOP-lowering and neuroprotective benefits of omega-3 fatty acids (Table 1). These inconsistencies likely reflect heterogeneity in study design, dosage, treatment duration, formulation (phospholipid vs. triglyceride), and participant characteristics. Many reports rely on self-reported intake data or animal models with high interspecies variability. Furthermore, the influence of total PUFA load and background omega-6 intake may modulate prostaglandin-mediated IOP regulation in ways that differ across populations. Well-designed randomized controlled trials with standardized supplementation regimens, defined glaucoma subtypes, and longer follow-up are needed to clarify causality and determine optimal dietary or therapeutic recommendations.

## 9. Conclusions

High-fat dietary patterns affect ocular health through overlapping mechanisms involving disrupted lipid metabolism, chronic inflammation, oxidative stress, and mitochondrial dysfunction. While excessive saturated fat intake consistently correlates with retinal degeneration, dry eye, and cataract formation, the role of unsaturated and polyunsaturated fats is more nuanced. Omega-3 fatty acids (EPA and DHA) show potential benefits for retinal integrity, tear film stability, and intraocular pressure regulation, but results across studies remain heterogeneous. Collectively, current evidence supports maintaining a balanced omega-3/omega-6 intake and avoiding excessive total fat consumption to promote ocular resilience. However, much of the data derive from small or short-term studies, often lacking dietary standardization or mechanistic validation. Future research should prioritize well-controlled clinical trials, mechanistic studies of lipid signaling in ocular tissues, and integration of dietary biomarkers with imaging and functional outcomes to establish evidence-based nutritional guidelines for eye health.

## Figures and Tables

**Figure 3:**
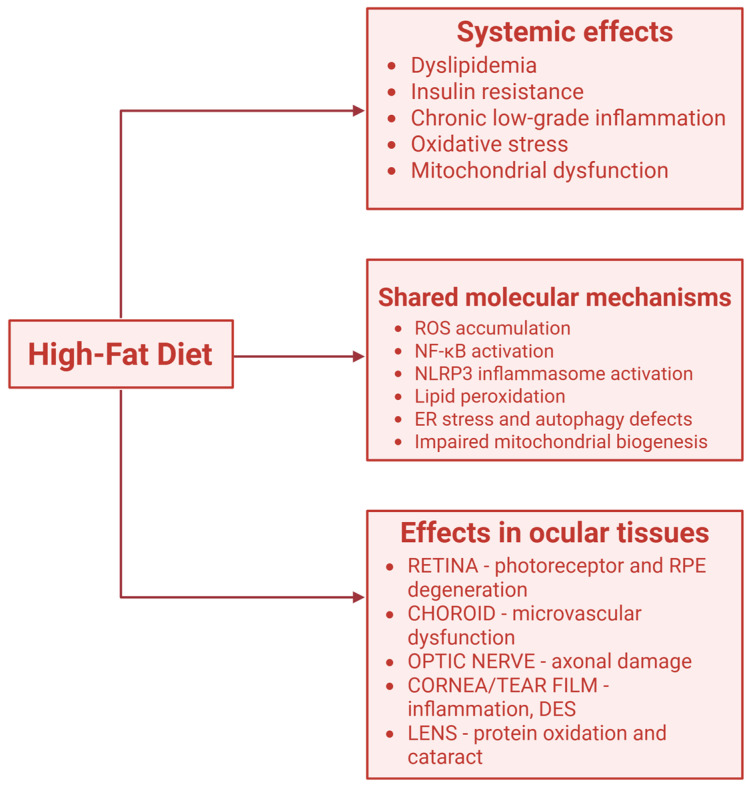
A summary of shared mechanisms of high fat diet on eye structures. Created in BioRender. https://BioRender.com/7zzxo8d (accessed on 12 October 2025).

**Table 1:** A summary of key clinical trials regarding omega-3 fatty acids supplementation.

Eye Condition	Study (Year)	Poulation/Design	Formulation and Dose	Duration	Primary Outcome	Main Findings
DES	Tellez-Vazquez (2016) [23]	Glaucoma patients with dry eye (*n* = 536), multicenter prospective trial	1680 mg EPA + 560 mg DHA per day (fish oil)	3 months	OSDI score, tear breakup time (TBUT)	Significant improvement in symptoms and TBUT
	Brignole-Baudouin et al. (2011) [27]	Dry eye (*n* = 145), double-masked RCT	Omega-3/6 blend (2 g/day)	6 months	Conjunctival HLA-DR expression	Reduced inflammatory markers
	Bhargava et al. (2023) [29]	Dry eye (*n* = 300), RCT	1000 mg omega-3/day	12 weeks	Schirmer test, OSDI	No significant clinical improvement
	Park et al. (2021) [30]	Post-cataract patients (*n* = 102), RCT	Re-esterified TG (rTG) omega-3, 2000 mg/day	8 weeks	TBUT, corneal staining	Improved tear stability and corneal integrity
	Hong et al. (2025) [31]	MGD after cataract surgery (*n* = 120), RCT	rTG omega-3 (1680 mg EPA + 560 mg DHA)	12 weeks	Lipid layer thickness, MG function	Improved meibomian gland function
Myopia	Pan et al. (2021) [14]	Experimental & clinical models	Dietary ω-3 PUFA	Variable	Axial length	ω-3 intake protective against myopia progression
	Zhang et al. (2025) [15]	Hong Kong Children Eye Study (*n* ≈ 4000), cross-sectional	Dietary ω-3 intake	-	Refractive error, axial length	Higher ω-3 intake associated with lower myopia risk
	Xue et al. (2024) [16]	Genomic MR and epidemiologic data	Genetic instrument for ω-3 PUFA	-	Myopia risk	ω-3 inversely associated with myopia prevalence
Glaucoma	Downie & Vingrys (2018) [59]	Healthy adults (*n* = 105), RCT	3000 mg omega-3/day	3 months	Intraocular pressure (IOP)	Reduced IOP by ~8%
	Luo et al. (2025) [62]	POAG patients (*n* = 80), RCT	Herring caviar oil (2000 mg EPA + DHA/day)	6 months	IOP, visual field	Modest IOP reduction and visual field trend
	Pérez de Arcelus et al. (2014) [52]	SUN cohort (*n* = 19,255), prospective	Dietary ω-3:ω-6 ratio	6.5 years	Incident glaucoma	Higher ω-3:ω-6 ratio linked to lower glaucoma risk
Age-Related Macular Degeneration/Retinal Health	Wu et al. (2017) [42]	Prospective cohort (*n* = 75,889)	Dietary EPA + DHA intake	12 years	AMD incidence	Higher intake associated with reduced AMD risk
	Schunck et al. (2018) [5]	Mechanistic review	-	-	Mechanistic insights	ω-3 metabolites (EETs) modulate inflammation, vascular tone

## Data Availability

No new data were created or analyzed in this study. Data sharing is not applicable to this article.
